# Young Children's Understanding of Teaching and Learning and Their Theory of Mind Development: A Causal Analysis from a Cross-Cultural Perspective

**DOI:** 10.3389/fpsyg.2017.00725

**Published:** 2017-05-16

**Authors:** Zhenlin Wang, X. Christine Wang, Wai Yip Chui

**Affiliations:** ^1^Department of Psychology, The Education University of Hong KongTai Po, Hong Kong; ^2^Department of Learning and Instruction, University at Buffalo, SUNYBuffalo, NY, USA; ^3^Assessment Research Centre, The Education University of Hong KongTai Po, Hong Kong

**Keywords:** theory of mind, teaching and learning comprehension, causality model, cross-cultural comparison, school readiness

## Abstract

Children's understanding of the concepts of teaching and learning is closely associated with their theory of mind (ToM) ability and vital for school readiness. This study aimed to develop and validate a Preschool Teaching and Learning Comprehension Index (PTLCI) across cultures and examine the causal relationship between children's comprehension of teaching and learning and their mental state understanding. Two hundred and twelve children from 3 to 6 years of age from Hong Kong and the United States participated in study. The results suggested strong construct validity of the PTLCI, and its measurement and structural equivalence within and across cultures. ToM and PTLCI were significantly correlated with a medium effect size, even after controlling for age, and language ability. Hong Kong children outperformed their American counterparts in both ToM and PTLCI. Competing structural equation models suggested that children's performance on the PTLCI causally predicted their ToM across countries.

## Introduction

In a trial that eventually led to his death, Socrates defended himself by claiming: All I know is that I know nothing. Amidst of the various readings of this Socrates' paradox, how we know that we (do not) know is the fundamental question from a personal epistemological perspective. During early childhood, personal epistemology undergoes qualitative developmental changes, and is associated with theory of mind (ToM) development (Burr and Hofer, 2002; Hofer, 2002). In this study, we aimed to develop and validate a scale of young children's understanding of the concepts of teaching and learning, an important dimension of personal epistemology (Ziv and Frye, 2004; Frye and Ziv, 2005; Ziv et al., 2008; Wang, 2010), and establish causality between young children's comprehension of teaching and learning and their ToM ability from a cross-cultural perspective. This line of inquiry can enrich our understanding of school readiness and pedagogy in early childhood.

### ToM and teaching and learning comprehension during early childhood

ToM is the awareness and reasoning of others' and one's own mental states (for a comprehensive review, see Hughes and Devine, 2015). Associated with cognitive correlates such as executive function and language, as well as social correlates such as family, peers, and cultural contexts, ToM development is evident from infancy to adolescence and most research shows that around the age of 4, children realize that people's behavior is guided by their beliefs that might be different from their own beliefs or the reality. Inherently related to knowledge formation, understanding how the mind works is closely associated with children's interpretation of the nature of teaching and learning (Olson and Bruner, 1996; Kuhn, 2000; Wellman and Lagattuta, 2004). Unfortunately, ToM research in developmental psychology predominately focuses on the acquisition of false belief understanding during early childhood (Wellman et al., 2001). As a result, our understanding of the relationship between ToM and children's comprehension of teaching and learning is limited.

Rooted in the inquiry of the nature of knowing and knowledge, personal epistemology explores people's thoughts on what knowledge is and how knowledge is constructed and evaluated (Burr and Hofer, 2002; Kuhn and Weinstock, 2002). Kuhn (1999) distinguished the levels of epistemological thinking before and after children achieve the false belief understanding as the realist level and the absolutist level. The realist level of epistemological thinking perceives people's assertions as copies of an external reality, whereas the absolutist level sees the assertions as either correct or incorrect representations of the reality. The transition from the realist level to the absolutist level is a decisive moment in the development of epistemological thinking. It indicates the “transition from simple, unconscious, unreflective knowing about the world to a second-order, or metacognitive, reflection on the knowing claims of self and others” (Kuhn and Weinstock, 2002, p. 126).

Understanding the mental process of teaching and learning is vital for becoming metacognitive self-regulated learners. By working definition, learning, and teaching in the current context refer to acquisition of explicit knowledge instead of tacit knowledge. Adopting a mentalisitic perspective, Ziv and Frye (2004) defined teaching as “an intentional activity to increase the knowledge (or understanding) of another, thereby reducing the knowledge difference between teacher and learner” (p. 458). Learning, on the other hand, is not necessarily intentional, although it entails knowledge change similar to that in teaching (Wang, 2010).

There is evidence that children's understanding of teaching and learning develops during preschool years, alongside with their emerging ToM ability (Ziv and Frye, 2004; Frye and Ziv, 2005; Ziv et al., 2008; Wang, 2010). Ziv and Frye (2004) developed stories of different teaching scenarios to probe children's understanding of the knowledge state in teaching. They found 3-year-old children responded that the ignorant person should be taught, and the knowledgeable person should teach, regardless of their other characteristics such as age or occupation status. However, when the teacher overestimated his or her own knowledge or the learner's knowledge, only 5-year-old children who had passed the false belief task predicted that the teacher's beliefs, but not the actual knowledge state, would dictate the teaching behavior. Children's performance on the teaching tasks involving teacher's false beliefs correlated with their performance on the false belief task, *r* = 0.31, controlling for age. This result was since replicated in French children, with a correlation between teaching stories involving false belief and ToM at 0.23 (Bensalah et al., 2012).

Children's performance on false belief tasks was also found to be related with their understanding of teaching intentions. In another series of studies (Frye and Ziv, 2005; Ziv et al., 2008), children were told stories either involving a teaching intention or not, and asked to judge whether the teacher intended to teach. For example, in the successful imitation story, the teacher was unaware of the presence of the learner, hence no teaching intention; in the embedded teaching story, the teacher disguised her teaching as playing a game instead of explicitly labeling it as an intentional teaching event. Again, only 5-year-old children could distinguish the teaching intention from the learning intention in the imitation story, or detecting a teaching intention embedded in a game. The understanding of teaching intentions was correlated with children's false belief understanding after the effect of age was partialed out, with *r* ranging from 0.33 to 0.59 across various tasks.

Focusing on the concept of learning, Wang (2010) developed a set of stories to examine the relation between ToM development and children's understanding of knowledge change and intention in learning. It was demonstrated that children's understanding of the learning concept underwent a transformation from a behaviorist perspective to a mentalistic perspective from 4- to 6-years of age. In the coincidence story for example, one protagonist was told that the circle she drew was exactly the letter *O*, while the other protagonist was simply complemented what a good job he did in drawing the circle. Children were asked to judge which one of the two characters learned how to write a letter *O* in the story. The results showed that young children responded in random, indicating a behaviorist view of learning. Similar to teaching comprehension, they also failed to appreciate the effect of false belief on learning. When the learner overestimated or underestimated own knowledge, younger children found it difficult to decide whether the learner needed to learn, or whether the learner would try to learn. Younger children also encountered difficulty in understanding learning stories with a conflict between the learning intention and the learning outcome. They over-attributed learning intentions to learners who learned something either by accident (hence a discovery learning) or through implicit learning without an initial intention. Changes in children's understanding of learning were correlated with their emerging ToM ability, with partial correlations ranging from 0.24 to 0.36 controlling for age across different tasks.

The small to medium effect sizes of the association between ToM and teaching and learning comprehension reviewed above suggested that these are two distinctive yet closely related constructs. The relation could be bidirectional: on the one hand, advanced mental state reasoning might help children to process teacher's intention and reflect on their learning; on the other hand, children's conceptualization of teaching and learning from everyday experience, especially that in formal schooling context, might provide children with more opportunities to discuss their mental state and practice mental state reasoning, hence lead to more advanced ToM.

This latter view is supported by the social perspective of ToM (Hughes and Devine, 2015) that emphasizes the environmental influence on mental state reasoning development. In the last two decades, various lines of inquiry have demonstrated that social input enriched with mental state discourse advances children's ToM development. Such inquiries include mental state discourse training in classrooms (e.g., Lecce et al., 2014), conversational environment of deaf children (e.g., Peterson and Siegal, 1995), and family characteristics including maternal sensitivity (e.g., Meins et al., 2003), content, and quality of family conversation (e.g., Jenkins et al., 2003), and sibling relationship (e.g., Hughes et al., 2010). In the case of schooling experience, Flavell (1987) claimed 30 years ago that the epistemic discourse in teaching and learning context could be a “hotbed” (p. 27) for the acquisition of false belief understanding. Surprisingly, little empirical evidence exists to date demonstrating how and to what extent the formal schooling affects children's ToM development, with the exception of some indirect indications. For example, it is suggested that early access to formal schooling might lead to more advanced mental state understanding (e.g., Bensalah et al., 2012). Indeed, cross-cultural comparison found British children who started formal schooling at an early age outperformed Italian and Japanese children on false belief understanding (Hughes et al., 2014). A more recent cross-cultural study demonstrated that comparing to British children, Hong Kong children attending local primary schools which predominately adopted the drill-and-practice pedagogy demonstrated delay in ToM use, while children attending international schools in Hong Kong adopting an inquiry based pedagogy were not delayed (Wang et al., 2016), even though in both local schools and international schools Hong Kong children outperformed their British counterparts in executive function, a cognitive stipulation for ToM (Devine and Hughes, 2014). The finding hinted the quality of epistemic discourse in schools plays an important role in children's ToM use in middle childhood.

Therefore, the question boils down to whether early comprehension of teaching and learning affects children's ToM development, and to what extent. The primary goal of the current study is to empirically test the relationship between children's teaching and learning comprehension and their ToM development by consolidating and validating a comprehensive teaching and learning scale for young children based on previous studies. More importantly, the current study goes beyond previous research and conducts causality analysis based on empirical data to answer the direction of causality question in the relation between teaching and learning comprehension and children's ToM ability. In order to achieve this goal, however, it is necessary to consider the cultural context of both ToM development and teaching and learning comprehension. Modern schooling as a rather recent phenomenon in our evolutional history functions as an important, if not the most important, institution for cultural transmission (Bjorklund and Bering, 2002). Schooling experience as a form of social input is undeniably culturally specific (Gutiérrez and Rogoff, 2003). Hence our conceptualization of teaching and learning is a product of both cultural beliefs and schooling practice.

### ToM and teaching and learning comprehension in cultural context

Both ToM and teaching and learning comprehension are arguably developed in cultural context. Even though children from different countries develop false belief understanding between 3- and 5-years of age (Wellman et al., 2001), there are remarkable cultural differences in the rate and trajectory of ToM development during early childhood (e.g., Naito and Koyama, 2006; Wellman et al., 2006; Liu et al., 2008; Shahaeian et al., 2011; Hughes et al., 2017). Both Japanese children (Naito and Koyama, 2006) and Hong Kong children (Liu et al., 2008; Hughes et al., 2017) were reported to show delayed false belief understanding acquisition comparing to Western children. Chinese (Wellman et al., 2006) and Iranian (Shahaeian et al., 2011) children develop knowledge understanding prior to belief understanding measured by a ToM scale, contrary to that of North American and Australian children.

Conceptualization of teaching and learning could be culture specific depending on cultural belief and context. Explicit knowledge transmission especially in the formal schooling system was not always the norm. Rogoff et al. (1993) reported that Mayan children rely exclusively on observing more experienced adults to learn. In such an observational learning context, it is probably justifiable to conceptualize learning as *doing* instead of *knowing*. In another light, while learning means *acquiring knowledge and understanding* in Kantian philosophy, Li (2001, 2002) argues that learning in Confucius philosophy implies virtue, or *perfecting oneself*, in addition to its epistemic nature. Supporting this view, Wang (2010) found that Chinese children insisted on the person who already learned certain knowledge should keep on “learning” the same knowledge again and again for the purpose of practice and achieving perfection.

In addition to the virtuous nature of learning, Asian cultures, especially Chinese cultures, highly value persistence and effort in children's learning. Asian children spend longer time in schools (Stevenson et al., 1990), and longer hours doing homework (Chen and Stevenson, 1989). Comparing to that in the West where formal academic learning usually starts around the age of five and six or even later, early childhood education in Hong Kong is heavily influenced by formal education practice (Pearson and Rao, 2006). Hong Kong children start formal numeracy and literacy learning as early as 3 years of age in kindergartens, often with daily homework (Li and Rao, 2000, 2005; Ng, 2014; Ho et al., 2015). As many of 81% of the kindergartens in Hong Kong teach two-digit addition, 42% of them teach two digit addition with carry-over (Cheng et al., 2001). A recent survey showed that 9-year-old Hong Kong primary school children on average spend 151 min every weekday doing homework (Ho, 2016). At home, Chinese parents are more involved in their children's learning, and demonstrate more formal teaching methods in their parenting (Pomerantz et al., 2014). Hong Kong government started to provide early childhood education vouchers to families since 2006, yet middle class parents tend to spend the extra cash to pay for extracurricular academic learning (Rao et al., 2016).

In sum, growing up in an intensely academic-oriented society, Hong Kong children are more likely to have a rather mature understanding of teaching and learning from an early age. This inspires another goal of the current study, which is to compare children's epistemic beliefs in the context of teaching and learning across cultures, in this case, Hong Kong and the United States. Going beyond the WEIRD (Western, Educated, Industrialized, Rich, and Democratic) societies that the majority of the research is based on, this is the first study of its kind examining cross-cultural differences in children's comprehension of teaching and learning, as well as the relation between teaching and learning comprehension and ToM development in a cultural context. The result would inform us the similarities and differences in children's personal epistemology across cultures, and highlight the role of cultural belief and practice as social input in ToM development.

In summary, we set out to meet three aims in this study. The first aim was to develop and validate a comprehensive Preschool Teaching and Learning Comprehension Index (PTLCI) based on tasks derived from previous studies investigating children's understanding of knowledge and intention in teaching and learning (Ziv and Frye, 2004; Frye and Ziv, 2005; Ziv et al., 2008; Wang, 2010), and establish measurement and structural equivalence of the PTLCI with a multilevel structural equation model (MSEM) (Muthén, 1994; Byrne and van de Vijver, 2014) using samples drawn from the U.S. and Hong Kong. The second aim was to examine the correlation between children's teaching and learning comprehension, measured with PTLCI, and their ToM development using a multiple-indicator-multiple-cause structural equation model (MIMIC-SEM) (Kline, 2016). In doing so it also enables us to examine the cross-cultural differences in the PTLCI and ToM development. Last but not the least, the third aim was to empirically evaluate the relationship between ToM and teaching and learning understanding and identify causal relationship through the MIMIC-SEM and reverse causality analysis.

## Current study

### Participants

Two groups of 4–6-year-old children participated in this study. The U.S. sample was consisted of 97 English speaking children including 42 girls and 55 boys (*M* = 64.6 months, *SD* = 9.63), recruited through kindergartens and schools serving middle class neighborhood in a large East Coast city of the United States. The majority of the sample was Caucasian. Two third of the mothers in the American sample (*n* = 61) had university level or above education. The Hong Kong sample was consisted of 106 Cantonese speaking children from Hong Kong including 58 girls and 48 boys (*M* = 63.8 months, *SD* = 9.81), recruited through kindergartens and schools serving middle class neighborhood in the New Territories of Hong Kong. All children in the Hong Kong sample were ethnic Chinese. Less than half of the Hong Kong mothers (*n* = 43) had university level or above education. The study was approved by the Human Research Ethics Committee of the Education University of Hong Kong and the Institutional Review Board of University at Buffalo, respectively. Written informed consents were obtained from parents and guardians before the data collection.

### Measures and procedures

#### Preschool teaching and learning comprehension index (PTLCI)

The PTLCI was comprised with 16 teaching and learning understanding tasks (Table 1, for story contents, see Appendix A in Supplementary Material) adapted from Frye and Ziv (2005), Ziv and Frye (2004), and Wang (2010). Each story described a teaching or learning scenario, concerning either knowledge state or teaching or learning intention. Both the teaching stories and the learning stories included four knowledge state stories and four intention stories. A task question was designed at the end of each story probing children's understanding of the learning scenario. The stories were told in a semi-random sequence with two of easiest stories, Who should be taught and Who learned, always at the beginning. The stories were told with prompts including Lego people and various objects and pictures demonstrating the teaching and learning scenarios.

**Table 1:** **Item parcel particulars of the 16 PTLCI items**.

**Parcels**	**Items parceled**
Parcel 1: Knowledge in teaching	Who should be taught Who can teach Overestimate learner's knowledge Overestimate own knowledge
Parcel 2: Teaching intention	Successful teaching Successful imitation Failed teaching Embedded teaching
Parcel 3: Knowledge in learning	Who learned Previous learning Coincidence False belief and ignorance
Parcel 4: Learning intention	Successful learning Failed learning Discovery learning Implicit learning

#### Theory of mind (ToM)

ToM was measured using two tasks drawn from Wellman and Liu's (2004) ToM scale, i.e., the *Knowledge Access task* and the *Content False-Belief task*. The *Knowledge Access task* started with the experimenter showing the child a small nondescript rectangular container with a single drawer. The child was asked what he or she thought was inside the drawer. The experimenter then pulled the drawer to reveal it was in fact a toy dog in it. The child was asked to confirm the real content of the drawer. Afterwards the child was introduced to a figurine with the name of Polly, and told Polly had never seen the inside of this drawer. The test question was whether Polly knew what was in the drawer. In the end the child was asked a memory control question of whether Polly had seen the inside of the drawer. To be scored correct the child must answer the target question “No” and answer the memory control question “No.”

The *Content False-Belief task* shared a similar format with the *Knowledge Access Task*, with a standard Band-aid box replacing the nondescript drawer. The child was prompt to answer *Band-aid* when asked about the content of the box before the real content, in this case a toy pig, was revealed. Other procedures and questions were similar to that in the *Knowledge Access Task*.

#### Receptive language ability

Children's language ability predicts ToM development (Milligan et al., 2007) and is intimately related to children's teaching and learning experience in school. In this study, receptive language ability was measured using Peabody Pictorial Vocabulary Test (PPVT-IV). On each trial, the examiner read aloud a word and the children were required to point to one of four pictures that provided the best match for that word. Adopting a back-translation approach (Brislin, 1970), each word in PPVT-IV was translated into Cantonese and then back-translated into English by a panel of three English/Cantonese bilingual developmental psychologists that included the lead author. The panel discussed and modified the Cantonese version to ensure that the two versions were equivalent in meaning. The Cantonese version was checked against *Lexical items with English explanations for fundamental Chinese learning in Hong Kong schools* provided by the Hong Kong Education Bureau (2009) to ensure progressive difficulty of the vocabulary. We calculated the raw scores by subtracting the number of errors made by each participant from the item number corresponding to the last number in the participant's ceiling set.

At each site, all tests were conducted on an individual bases in a quiet room located in the participants' schools or in a research lab. The sequence of the tasks was counterbalanced. The testing session lasted for about 1 h. Children were free to take rest breaks during the testing session if they chose to.

### Analytic approach: multilevel-multicultural perspective

To validate a measurement universally adaptive and generalizable in both the and Hong Kong culture at the sample level as well as at the country level, we adopted the multilevel-multicultural perspective (Byrne and van de Vijver, 2014) which could address the disaggregated individual and aggregated cultural data to establish measurement equivalence and structural equivalence. Measurement equivalence refers to the equality of the mean of the manifest variable; while structural equivalence means the equality of the relationships among latent variables. By adopting a latent variable approach in contrast to the manifest variable approach (Selig et al., 2008), we aimed to decompose the total variance into within- and between-group components and enhance specifying separate structural model at each level (Byrne and van de Vijver, 2014).

Step 1 would be the construct validation of the PTLCI by confirmatory factor analysis (CFA) using item-parceling to reduce the number of parameters in order to improve model fit and decrease noises in investigating the structural models (Bandalos, 2002; Little et al., 2002).

Step 2 would establish a multilevel structural equation model (MSEM) (Muthén, 1994; Byrne and van de Vijver, 2014) to examine the measurement and structural equivalence across the country and the individual levels.

Step 3 and Step 4 would evaluate the relationship between ToM and teaching and learning understanding and identify causal relationship through reverse causality analysis. In Step 3, we adopted a multiple-indicator-multiple-cause structural equation model (MIMIC-SEM) (Kline, 2016). MIMIC-modeling is an integration of both formative and reflective modeling. In a formative model, observed variables are the causes of the latent variables. On the contrary, in a reflective model, latent variables are the causes of the observed variables. Hence, in a MIMIC-structural model, the independent observed variables serve as the causes of the latent variables which in turn contribute to the outcome dependent observed variables. Step 4 would target at ensuring causality of the structural models using reversed causality analysis. In a cross-sectional design, reversed causality analysis will confirm the directionalities of causations among the studied constructs by reversing the direction of causality and comparing the model fitness indexes to that of the original model. The model with significantly better model fitness would be accepted as the causality model (Antonakis et al., 2010).

## Results

We conducted the analysis based on maximum likelihood estimates using LISREL 9.2 (Kline, 2016). The criteria for good model-fitness were comparative-fit index *(CFI)* ≥0.95, goodness-of-fit index *(GFI)* ≥0.90, root mean square error of approximation *(RMSEA)* ≤0.08, and standardized root mean square residual *(SRMR)* ≤0.08 (Byrne, 1998; Hu and Bentler, 1999; Kline, 2016).

### Step 1

The analyses were composed of two phases. First, in order to ensure the construct validity of the PTLCI, the relationships among the items and the latent factors were evaluated by confirmatory factor analyses (CFAs) by means of maximum likelihood (ML) estimation. The rationale for item parceling was based on the face validity of the 16 PTLCI (Marsh et al., 1999). This step would be conducive to investigating and ameliorating the potential measurement problems. The item parcels of the 16 items were illustrated by Table 1 above.

Prior to the CFAs, multivariate and univariate data screening had been conducted involving the investigating outliers, skewness, and kurtosis for the sake of the normality assumption of CFA, although with a sample size larger than 200, the problem of skewness and kurtosis was not a major concern (Tabachnick and Fidell, 2001). The skewnesses of all the items fell between −2 and 2 while the kurtoses of all the items fell between −7 and 7 (George and Mallery, 2010; Table 2), hence all of the data were retained.

**Table 2:** **The skewnesses and kurtoses of all the variables within country and of the entire dataset**.

	**Entire dataset**		**Hong Kong**		**U.S**.	
	**Skewness**	**Kurtosis**	**Skewness**	**Kurtosis**	**Skewness**	**Kurtosis**
Age	−0.022	−1.007	−0.073	−1.041	0.041	−0.986
PPVT	−0.332	0.099	−0.005	0.203	−0.705	0.410
PTLCI P1	−0.355	−0.207	−0.490	−0.572	−0.277	0.389
PTLCI P2	−0.371	−0.758	−0.361	−0.638	−0.316	−0.945
PTLCI P3	−0.769	0.315	−0.713	−0.277	−0.685	0.306
PTLCI P4	−0.017	0.036	0.165	0.615	−0.039	−0.428
ToM P1	−0.370	−1.264	−0.419	−1.110	−0.313	−1.416

The means, *SDs* and the intercorrelations of and among all the studied variables were tabulated by Table 3 below.

**Table 3:** **The descriptive statistics and intercorrelations of all the variables studied**.

	**Mean**	**SD**	**1**	**2**	**3**	**4**	**5**	**6**	**7**
1. Age	5.35	0.81	1.00						
2. PPVT	99.46	14.02	0.01	1.00					
3. PTLCI P1	0.70	0.22	0.26^***^	0.12	1.00				
4. PTLCI P2	0.82	0.17	0.25^***^	0.13	0.23^**^	1.00			
5. PTLCI P3	0.79	0.21	0.22^**^	0.13	0.22^**^	0.15^*^	1.00		
6. PTLCI P4	0.05	0.23	0.18^*^	0.09	0.11	0.15^*^	0.07	1.00	
7. ToM P1	1.20	0.78	0.43^**^	0.20^**^	0.16^*^	0.14^*^	0.20^**^	0.21^**^	1.00

* p < 0.05;

** p < 0.01;

*** *p < 0.001*.

**Figure 1:**
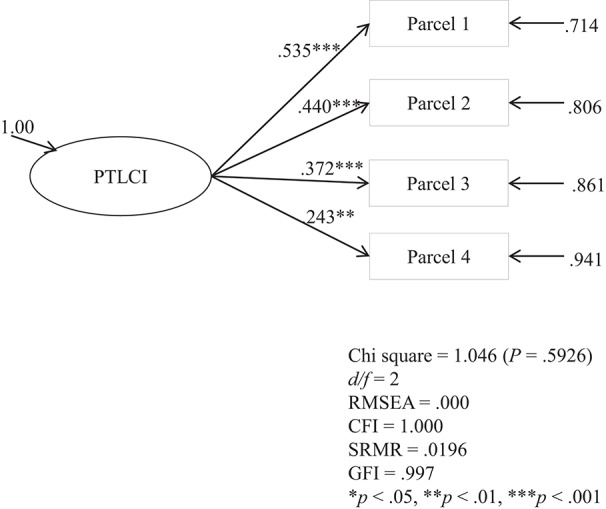
**Confirmatory Factor Analysis of the 4-parcel model of the PTLCI**.

The CFA result indicated that the 4-parcel model of the PTLCI fit the data sufficiently: χ^2^ (*df* = 2) = 1.046, *p* = 0.5926, *CFI* = 1.00, *SRMR* = 0.0196, *GFI* = 0.997, *RMSEA* = 0.000. The result demonstrated excellent construct validity of the PTLCI. The aforementioned CFA model is depicted by Figure 1 below.

### Step 2

**Figure 2:**
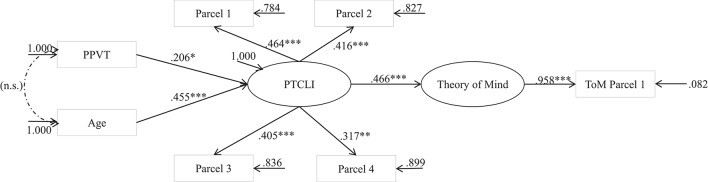
**The MSEM between-country/within-country structural model of PTLCI**. ^*^p < 0.05, ^**^p < 0.01, ^***^p < 0.001.

The focus of Step 2 was to establish measurement and structural equivalence of the PTLCI between and within each country. The MSEM analytical strategies purported by Muthén (1994) and Byrne and van de Vijver (2014) were employed in this study. A two-level Structural Equation Model (SEM) was adopted to include the between country level and the within country (individual) level, taking age, and language ability into consideration. As mentioned above, in MSEM, the overall variance could be decomposed to the group and individual levels. The between-country/within-country structural model was depicted by Figure 2 below, with good model fit: χ^2^ (*df* = 32) = 20.200, *p* = 0.9477, *CFI* = 1.00, *SRMR* = 0.0343, *GFI* = 0.986, *RMSEA* = 0.000. Therefore, the aforementioned multilevel model as well as the structural equivalence were duly validated across both the country and individual levels. It was implied that the PTLCI as well as the ToM measure were both culturally adaptive in both Hong Kong and the U.S. Furthermore, in both regions, a child's PTLCI score would have a significant and positive contribution to the child's ToM capacity, with a standardized effect size of 0.466.

### Step 3

**Figure 3:**
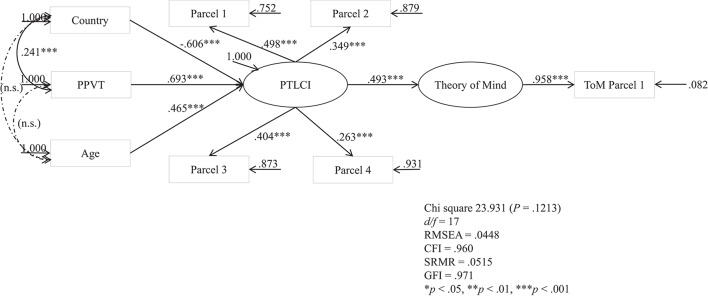
**The MIMIC-SEM model of PTLCI**.

The focus of Step 3 was 2-fold: one, to capitalize on the association between PTLCI and ToM found in Step 2 using a MIMIC-SEM to determine causality; and two, to examine cross-cultural differences of the two constructs of interests by entering country in the model. The MIMIC-SEM result was illustrated in Figure 3. The model indicated that the hypothetical model fit the data sufficiently: χ^2^ (*df* = 17) = 23.931, *p* = 0.1213, CFI = 0.960, SRMR = 0.0515, GFI = 0.971, RMSEA = 0.0448. The standardized indirect effect of the independent variables were illustrated by Table 4 below. The MIMIC structural model indicated that the children in Hong Kong outperformed their U.S. counterparts in the PTLCI (β = −0.606, *p* < 0.001). Moreover, according to the Table 4, Hong Kong children also outperformed their U.S. counterparts in ToM, with standardized indirect effect = −0.299, *p* < 0.001.

**Table 4:** **The standardized indirect effects of the independent observed variables on the dependent latent variables in the MIMIC-model**.

	**Country**	**Age**	**PPVT**
ToM	−0.299^***^	0.342^***^	0.230^***^

^*^p < 0.05; ^**^p < 0.01;

*** *p < 0.001*.

### Step 4

In order to investigate the causalities of the models, reversed causality analyses were conducted in this study. The results were illustrated by Table 5 below.

**Table 5:** **The differences among the goodness-of-fit indices of the validated and the reversed causality models**.

**Model**	***χ^2^***	***d/f***	**RMSEA**	**CFI**	**SRMR**	**GFI**
MSEM	20.200	32	0.000	1.000	0.0343	0.986
MSEM-Reversed	55.355	32	0.060	0.914	0.0655	0.962
MIMIC-SEM	23.931	17	0.045	0.960	0.0515	0.971
MIMIC-SEM-Reversed	86.608	17	0.0142	0.600	0.0928	0.913
	Δ*χ^2^*	Δ***d/f***	Δ**RMSEA**	Δ**CFI**	Δ**SRMR**	Δ**GFI**
MSEM	35.155	0	0.060	0.086	0.0312	0.024
MIMIC-SEM	62.677	0	0.097	0.360	0.0413	0.058

Among the validated and reversed structural models, ΔCFI ≥ 0.01 which reflected that the validated and reversed models were not equivalent (Cheung and Rensvold, 2002). Under the comparison of CFI, GFI, SRMR, and RMSEA, the initially validated models demonstrated more desirable model-fit indices and hence was found to be more appropriate in terms of causality.

## Discussion

Our first aim was to develop and validate a comprehensive measure for young children's teaching and learning comprehension across cultures. The result from CFA of item parceling based on items' face validity generated a PTLCI model that sufficiently fit the data. Furthermore, measurement and structural equivalence was achieved using a two-level structural equation model assessing both the between country level and the within country (individual) level variances. Both the PTLCI and the ToM measure were culturally adaptive in Hong Kong and the United States. Our second aim was to examine the cross-cultural differences in the children's teaching and learning comprehension and ToM development, as well as the correlation between the two constructs in both cultures. Contrary to the previously reported delay in ToM comparing to Western children (Liu et al., 2008; Hughes et al., 2017), Hong Kong children outperformed their U.S. counterparts on both ToM measure, with a standardized indirect effect of −0.299, and PTLCI, β = −0.606. In both regions, the PTLCI score had a significant and positive contribution to the ToM capacity, with a moderate standardized effect size of 0.466. The third aim was to identify causal relationship between children's teaching and learning comprehension. Through MIMIC-SEM and reverse causality analysis, we demonstrated the model indicating that children's performance on the PTLCI causally predicted their ToM across countries was a better fit than the reverse model, providing empirical evidence for the direction of causality.

The results contributed to the understanding of young children's epistemic beliefs in several aspects. First, we replicated and consolidated previous reports on the association between false belief understanding and children's teaching and learning comprehension (Ziv and Frye, 2004; Frye and Ziv, 2005; Wang, 2010). Adopting a more comprehensive set of tasks on teaching and learning comprehension, we demonstrated a medium effect size association between young children's teaching and learning comprehension and their developing ToM ability, controlling for age and language ability. This result broadens the scope of ToM research in early childhood and provides the much needed evidence for the link between ToM development and academic readiness long suspected by scholars in the field (Astington, 1998; Astington and Pelletier, 2005).

Second, a noteworthy contribution of this study is the culturally adaptive PTLCI that can be used as a standalone measure of children's epistemic beliefs in the context of teaching and learning during early childhood. Further information about the materials and procedures are available upon request. Although the conceptualizations of teaching and learning potentially have certain cultural bearing, the epistemic nature of motivation and knowledge change is the essential core of teaching and learning. Our cross-cultural evidence demonstrates that as early as preschool period, children in both Hong Kong and the U.S. share common conceptualization of academic activities. This line of research adds to the current understanding that despite of differences in cultural beliefs and applications, common core values and good practices propel children's development and learning universally (Pomerantz et al., 2014; Hughes et al., 2017). The cross-culturally validated PTLCI is a psychometrically robust measure for preschool children's understanding of teaching and learning. It provides an easy-to-use measurement tool for teachers to assess individual child's teaching and learning understanding in a classroom setting in order to adapt pedagogy and curricula to accommodate each child's metacognitive developmental level. Schools could potentially use the PTLCI as a school readiness assessment for that metacognitive self-regulation has been identified as one of the predictors of academic success in primary schools (e.g., Blair, 2002). In recent years studies on the long lasting effect of early epistemic beliefs have caught much attention. For example, a study found understanding of teaching and learning facilitates children's self-regulation (Porath et al., 2010). A cross lagged design found that earlier cognitive mental state knowledge predicts later metaknowledge about reading (Lecce et al., 2010). Future research linking young children's teaching and learning comprehension with school readiness and later academic achievement should be fruitful.

The third contribution of the current study is to lend support to the social view of ToM (Hughes and Devine, 2015) by highlighting the causal role of epistemic beliefs in the teaching and learning context on ToM development. Discourse in the context of teaching and learning, especially that used in inquiry based pedagogy, taps on knowledge, memory, belief, teaching and learning intention on a daily bases. Such exposure should be able to help children understand how learning comes about, which in turn facilitates their mental state understanding in general. This is the first empirical evidence to our knowledge to date demonstrating direction of causality between these two constructs. This result complements the studies demonstrating family and peer input on ToM development (Hughes and Devine, 2015) and highlights the importance of schooling experience in mental state reasoning development (Wang et al., 2016). Wang et al. (2016) argued for a pedagogical account based on their evidence on cross-cultural differences in ToM development in middle childhood, while the current study demonstrates that even before children enter formal schooling the conceptualization of teaching and learning is causally linked with their ToM capacity. The implication of such causality is that parents and early childhood teachers should engage children in discourse enriched in mental state reasoning. This is not to say we need to push even more academic learning into early childhood than there is already, it rather indicates that both teachers and parents need to be sensitive to children's beliefs and desires in their interaction and teaching. From this viewpoint, the mind-mindedness concept that advocates treating children as mental agents with their own beliefs and desires (Meins et al., 2003) should be extended to the pedagogical context for both parents and teachers. Future research should seek to directly test the effect of schooling experience on ToM development using longitudinal or intervention design.

Hong Kong children's more pronounced advantage in teaching and learning comprehension comparing to the American children corresponds with their earlier and more intense exposure in epistemic discourse. This might also explain Hong Kong children's small advantage on ToM measures considering the causal relationship between the two constructs. The discrepancy between the current results and Hong Kong children's reported delay in ToM (Liu et al., 2008; Hughes et al., 2017) might be attributed to the sampling bias, yet our statistical model controlled for relevant variables including age and language ability. More importantly, the direction of group difference on ToM does not affect our primary goals in this study, which was to validate the PTLCI and establish its causal role in ToM development across cultures. That being said, the culturally specific pathways of epistemic beliefs is still a rather under investigated area that warrants further examination.

## Ethics statement

This study was carried out in accordance with the recommendations of The Education University of Hong Kong's (EdUHK) Guidelines on Ethics in Research, and University at Buffalo's (UB) Institutional Review Board with written informed consent from guardians of all subjects. Guardians of all subjects gave written informed consent in accordance with the Declaration of Helsinki. The protocol was approved by EdUHK's Human Research Ethics Committee, and UB's Social and Behavioral Research Office.

## Author contributions

ZW contributed to the conceptualization of the manuscript, data collection in Hong Kong, and the preparation of the manuscript; XW consulted on the conceptualization of the manuscripts, and contributed on the data collection in the US; WC contributed to the data analysis of the manuscript.

## Funding

Funding for this study was partially provided by Internal Research Grant (Ref: 10107) from The Education University of Hong Kong to ZW.

### Conflict of interest statement

The authors declare that the research was conducted in the absence of any commercial or financial relationships that could be construed as a potential conflict of interest.
