# Supplementary material for: Young Children's Understanding of Teaching and Learning and Their Theory of Mind Development: A Causal Analysis from a Cross-Cultural Perspective
Source: Front Psychol. 2017 May 16;8:725. doi: 10.3389/fpsyg.2017.00725 (PMC5432649; doi:10.3389/fpsyg.2017.00725)
Supplement: Supplementary file 1 [file AppendixA.DOCX]

Appendix A: Preschool Teaching and Learning Comprehension Index (PTLCI)

***Knowledge state in teaching***

**T1. Who should be taught.**

Sally knows how to read. Andy does not know how to read. This is Ms. O, the teacher. She teaches children how to read. Who should Mrs. O teach how to read, Sally or Andy?

**T2. Who can teach, can the teacher be taught.**

One day Isabella comes to school and sees a new computer game. She does not know how to play the new game. The teacher does not know how to play the game. Isabella and Kevin are friends. Kevin knows how to play. Who will teach Isabella how to play the game? The teacher, or Kevin?

**T3. Overestimating learner’s knowledge.**

Ms. O, the teacher, teaches children how to write their names. Hillary does not know how to write her name, but Ms. O thinks that Hillary knows how to write her name. Does Hillary really know how to write her name or not? Right, Hillary doesn’t know how to write her name, but Ms. O thinks that she knows. What will Ms. O do? Will she try to teach Hillary how to write her name or not?

**T4. Overestimating own knowledge.**

Reese thinks he really knows how to count. This is how Reese counts: “7, 3, 20.” Reese thinks this is the right way to count. His kid sister doesn’t know how to count. Does Reese really know how to count or not? Right, Reese doesn’t know how to count but he thinks that this is the right way to count. Will Reese try to teach his kid sister how to count or not?

***Teaching intention***

**T5. Successful teaching.**

Jason does not know how to make a bowl from clay. Anna knows how to make a bowl from clay. Every day Anna shows Jason how to make a bowl from clay, so that he learns how to do it. “Look, first you roll the clay to a ball, then you stick your thumb into the ball, there, you have a bowl.” Now Jason also knows how to make a bowl. Did Anna try to teach Jason how to make a bowl from clay or not?

**T6. Successful imitation.**

Dave does not know how to tie his shoe laces. Lily knows how to tie shoe laces. Every day Dave watches Lily when she ties her shoe laces. See, he sits here, behind the table, watches Lily, and tries to do just what she does. Lily does not see Dave. Lily does not know Dave is watching her. Now Dave knows how to tie his shoelaces. Did Lily try to teach Dave how to tie his shoelaces or not?

**T7. Failed teaching.**

Gail does not know how to play Animal Dominos. Randy knows how to play Animal Dominos. Every day Randy teaches Gail how to play Animal Dominos, ‘‘See, first you look for the same picture as here, and then you put the picture right next to this one. There, now you have two same pictures right next to each other.’’ Gail tried and tried, but she still does not know how to play. Did Randy try to teach Gail how to play Animal Dominos or not?

**T8. Embedded teaching**.

Every day Ms. Naomi, the teacher, teaches kids in the class how to read numbers. Today, Ms. Naomi brings a game to class and says: “We are going to play the number game now. In this game every child gets a card and has to say what number appears on the card. The winner is the one who reads all the numbers.” When Ms. Naomi plays the number game with the kids, what does she really want? Does she want to play with the game with the kids, or does she really want to teach them numbers?

***Knowledge state in learning***

**L1. Who learned.**

Andrew and Betty do not know how to draw a square. This is Ms. Cindy. She is their teacher. Every day, Ms. Cindy shows them how to draw a square. Now Andrew knows draw a square. But Betty still does not know how to draw a square. Who learned to draw a square, Andrew or Betty?

**L2. Previous Learning.**

Evan knows how to count to 3. He learned that a long time ago. Today in school Flora asks Evan: “Do you know how to count to 3?” Evan says: “Yes, I do”. He counts like this: “1, 2, 3.” Did Evan learn how to count to 3 today, or did he learn that a long time ago?

**L3. Coincidence.**

Jack and Wendy do not know how to write the letter O. Today in school, Jack and Wendy each draw a circle. It looks just like the letter O. Jack shows it to the teacher. The teacher says to Jack: “This is a circle, you just drew a nice circle.” Jack still does not know this is how you write the letter O. Wendy shows her circle to the teacher. The teacher says to Wendy: “This is a letter O, you just wrote a letter O.” Now Wendy knows this is how you write the letter O. Who learned how to write a letter O today, Jack or Wendy?

**L4. False belief and ignorance.**

Here is a spoon. Winnie does not know what this is, she just does not know. Tim thinks he knows what this is, but actually he doesn’t. He calls it a fork. Who will try to learn what this is, Winnie or Tim?

***Learning intention***

**L5. Successful learning.**

Nina does not know how to find 3 o'clock on the clock dial. Will knows how to find 3 o'clock on the clock dial. Today in school, Nina watches Will find 3 o'clock on the clock dial. Will says: “When the long hand points to 12, and the short hand points to 3, it is 3 o’clock.” Nina watches really carefully and tries very hard to remember it. Now Nina knows how to find 3 o’clock on the clock dial. Did Nina try to learn how to find 3 o'clock on the clock dial or not?

**L6. Failed learning.**

Pit does not know all the colors in a rainbow. Kathy knows all the colors in a rainbow. Today in school, Pit watches Kathy name all the colors in a rainbow. Kathy says: “Red, orange, yellow, green, blue, indigo, and violet.” Pit watches really carefully and tries very hard to remember it. But Pit still does not know all the colors in the rainbow. Did Pit try to learn all the colors in the rainbow or not?

**L7. Discovery learning.**

Quincy does not know how to make green paint. Today in school, Quincy drops some blue paint in yellow paint by accident while painting. Oops! Look what happened. The two colors make green. “So that’s how you make green paint”, Quincy says. Quincy knows how to make green paint now. Did Quincy try to learn how to make green paint or did he not try?

**L8. Implicit learning.**

Rachel does not know how to sing the Birthday Song. Today in school, other kids are singing the Birthday Song. Rachel covers her ears and tries very hard not to listen to that song. But she can still hear their singing. Later when Rachel begins to sing, she sings the Birthday Song. Rachel knows how to sing the Birthday Song now. Did Rachel try to learn the birthday song, or did she try not to learn the Birthday Song?
